# Epstein–Barr Virus Reactivation as a New Predictor of Achieving Remission or Lupus Low Disease Activity State in Patients with Systemic Lupus Erythematosus with Cutaneous Involvement

**DOI:** 10.3390/ijms24076156

**Published:** 2023-03-24

**Authors:** Rada Miskovic, Andja Cirkovic, Danijela Miljanovic, Ivica Jeremic, Milka Grk, Milica Basaric, Ivana Lazarevic, Maja Stojanovic, Aleksandra Plavsic, Sanvila Raskovic, Ana Banko

**Affiliations:** 1 Clinic of Allergy and Immunology, University Clinical Center of Serbia, 11000 Belgrade, Serbia; 2 Internal medicine department, Faculty of Medicine, University of Belgrade, 11000 Belgrade, Serbia; 3 Institute for Medical Statistics and Informatics, Faculty of Medicine, University of Belgrade, 11000 Belgrade, Serbia; 4 Institute of Microbiology and Immunology, Faculty of Medicine, University of Belgrade, 11000 Belgrade, Serbia; 5 Institute of Rheumatology, 11000 Belgrade, Serbia; 6 Institute of Human Genetics, Faculty of Medicine, University of Belgrade, 11000 Belgrade, Serbia

**Keywords:** systemic lupus erythematosus (SLE), Epstein–Barr virus (EBV), predictor, SLE remission, lupus low disease activity state, anti-EA (D) IgM, biomarker, EBV reactivation

## Abstract

Although Epstein–Barr virus (EBV) reactivation has long been associated with the pathogenesis of systemic lupus erythematosus (SLE), many aspects of this relationship remain unclear. Our objective was to investigate the association between EBV reactivation and the achievement of SLE remission and lupus low disease activity state (LLDAS) over a six-month period. Clinical, laboratory, and virological tests (anti-EBV antibodies and EBV DNA) were performed among 51 patients with the active form of SLE on two occasions six months apart. SLE remission and LLDAS achievement were assessed at the end of the follow-up period. Active EBV infection was detected in 45% of active SLE patients at baseline, and 77% transitioned to latent EBV infection at six months (*p* < 0.001). Multivariate regression revealed a higher titer of anti-EA(D) IgM-Abs and the presence of anti-EA(D) IgM-Abs as independent predictors of remission and LLDAS in SLE patients with mucocutaneous manifestations (*p* = 0.042) and rash only (*p* = 0.023), respectively. Since a higher C3 level was an independent predictor of transition to latent EBV infection (*p* = 0.027), the estimated cut-off value that could identify active SLE patients who will transition to latent EBV infection after six months was ≥0.780 g/L with a sensitivity of 70.6% and a specificity of 75.0% (AUC = 0.756, *p* = 0.003). EBV reactivation is common in patients with active SLE, and most of them transition to latent EBV infection after six months. Achieving remission and LLDAS in SLE patients with mucocutaneous manifestations can be predicted by a higher titer, whereas in SLE patients who have only a rash, the presence of anti-EA (D) IgM-Abs was a predictor of remission and LLDAS.

## 1. Introduction

Systemic lupus erythematosus (SLE) is a systemic autoimmune disease characterized by a heterogeneous clinical presentation, autoantibody production, and a relapsing–remitting course in most patients. The pathogenesis of the disease is complex, and despite numerous efforts and studies, many aspects are still unknown [1]. Previous research has shown that type I interferons (IFN), produced mainly by activated plasmacytoid dendritic cells (pDCs), play an important role [2]. Increased IFN-α activity correlates with disease activity and its manifestations. In addition to various environmental factors, several infectious agents have been closely associated with the onset and progression of SLE in genetically susceptible individuals [3].

The Epstein–Barr virus (EBV) is a ubiquitous virus, lifelong present in nearly 95% of the world’s population. After primary lytic infection, the virus persists in a latent form in memory B-cells and uses various strategies to evade the immune system. During latency, only a limited number of genes are expressed. They encode proteins required for viral survival: six EBV nuclear antigens (EBNA-1, 2, 3A, 3B, 3C, and leader protein) and three latent membrane proteins (LMP1, 2A, and 2B) [4,5]. Occasional EBV reactivations are characterized by increased viral replication and expression of the lytic genes also [6]. The lytic genes encode a number of proteins required for viral replication and immune system evasion, such as diffuse early antigen (EA (D)), restricted early antigen (EA (R)), viral IL-10 homolog, viral capsid antigen (VCA), and membrane antigen (MA) [5].

In genetically predisposed individuals, EBV can cause immune dysregulation and the induction of autoimmunity. It has been described that the epitopes of the latent viral protein EBNA-1 cross react with several lupus autoantigens—C1q, SmB, SmD, and Ro—contributing to the development of lupus-specific autoimmunity via the mechanism of molecular mimicry [7,8]. The autoimmune process is further diversified through the process of epitope spreading. Several EBV proteins function as analogues of human proteins. The viral IL-10 homolog inhibits IFN-γ production and MHC-I expression, and promotes B-cell proliferation and differentiation. LMP1 acts as a functional homolog of CD40, and LMP2A mimics the B-cell receptor, promoting B-cell survival, and rescuing infected B-cells from apoptosis [9].

In addition, there is evidence of impaired control of EBV infection in SLE, leading to more frequent viral reactivations. SLE patients have reduced numbers of EBV-specific CD 8⁺ T-cells, impaired regulatory T-cell response, enhanced humoral immune response, and increased EBV viral load in the B-cells and the peripheral blood monocytes [10]. Impaired EBV infection control with frequent viral replication and subsequent immune activation could contribute to the exacerbation of SLE [11]. The Epstein–Barr virus can also trigger strong IFN-α production directly in pDCs, which further contributes to the systemic inflammation in SLE [12]. On the other hand, polyclonal B-cell activation during SLE flare may influence EBV reactivation, thus maintaining the vicious cycle. However, the nature of the relationship between EBV reactivation and SLE is not yet clear.

In order to explore the link between EBV reactivation and SLE activity, we examined markers of humoral immune response to lytic and latent EBV antigens and the presence of EBV DNA in a prospective cohort of patients with an active form of SLE over a six-month period.

## 2. Results

### 2.1. Characteristics of the Study Cohort

The study cohort consisted of 51 patients, predominantly women (94%), with a mean age of 42.49 (±13.06) years and a median disease duration of 5.00 (IQR 1.00–12.25) years. There were 20 (39%) patients with secondary Sjogren’s syndrome, 8 (16%) with autoimmune thyroiditis, and 3 (6%) patients with secondary antiphospholipid syndrome. Common nonautoimmune comorbidities were hypertension in 45%, cardiovascular events which include acute myocardial infarction, angina pectoris, cerebrovascular insult, and/or transient ischemic attack in 8%, and diabetes mellitus in 4% of patients. The mean number of fulfilled ACR 1997 criteria was six (4–11).

### 2.2. Clinical, Laboratory, and Therapeutic Characteristics of Patients during the Follow Up

A summary of the clinical, laboratory, and therapeutic characteristics of the study participants at baseline and six months of follow-up is shown in Table 1. Most patients had active mucocutaneous (78%), musculoskeletal (74%), and hematological (69%) manifestations that significantly decreased or completely resolved after the follow-up period and initiation of appropriate treatment. All disease activity scores (SLEDAI-2K and PGA) were significantly lower after follow up (*p* < 0.001 for both scores), with a median reduction in the SLEDAI-2K score for 6.0 (IQR 3.0–9.25) and the PGA score for 0.81 (IQR 0.45–1.23). A total SLEDAI-2K score ≤ 4 was achieved in 60% and a PGA score ≤ 1 in 82% of patients. The prednisone dose was reduced to ≤10 mg in 54% of patients. However, the damage index increased significantly.

### 2.3. EBV Infection Status during Follow Up

All SLE patients had evidence of previous exposure to EBV (100% anti-VCA IgG, 98% anti-EBNA-1 positive). To determine the status of EBV infection, the overall results of both molecular and serological tests were analyzed. Active EBV infection was detected in 45% of patients at baseline, with 77% of them transitioning to latent EBV infection at six months (*p* < 0.001). The transition from active to latent infection was not affected by therapy (prednisone dose, pulse corticosteroid therapy, use of immunosuppressants) or by the presence of other autoimmune diseases (Sjogren’s syndrome, antiphospholipid syndrome, and autoimmune thyroiditis). Of the 24 patients with active EBV infection detected at baseline, five were still chronically active after the follow-up period. In contrast to them, three (11.0%) patients developed active EBV infection after six months (Table 2). Transition to a latent EBV infection was not associated with the achievement of lupus remission and LLDAS (*p* = 0.308).

From eight (15.7%) samples positive for viral DNA at baseline, only one sample (2.0%) was still positive at six months (*p* = 0.219). In addition, one patient became EBV DNA positive after six months, although his result was negative at baseline.

**Figure 1:**
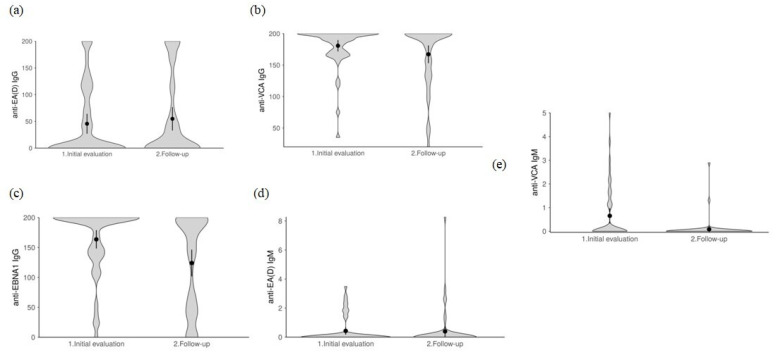
Violin plots for comparison of anti-EBV antibodies’ titer level during the follow up (**a**) anti-EA (D) IgG; (**b**) anti-VCA IgG; (**c**) anti-EBNA-1 IgG; (**d**) anti-EA (D) IgM; (**e**) anti-VCA IgM.

Analyzing EBV serological results, we found a significant reduction in seroprevalence for anti-EBNA-1 IgG and anti-VCA IgM after six months (*p* = 0.031 and *p* < 0.001, respectively) (Table 3). Of the 50 (98.0) patients who were positive for anti-EBNA-1 IgG at baseline, 42 remained positive and 6 became negative. Also, of the 16 (31%) patients who were positive for anti-VCA IgM at baseline, only two were positive after follow up, whereas none of the initially negative 35 (69%) changed their status after follow up. A comparison of anti-EBV antibody titers showed a significant decrease in anti-EBNA-1 IgG and anti-VCA IgM (*p* < 0.001 and *p* = 0.001, respectively) and an increase in anti-EA (D) IgG (*p* = 0.043) titers. There was no significant change in anti-EA (D) IgM and anti-VCA IgG titers (*p* = 0.778, *p* = 0.093, respectively) (Figure 1).

### 2.4. Presence of Anti-EA (D) IgM Antibodies in Patients with Active Rash Is Associated with Increased Chance of Achieving Remission and LLDAS

Considering that previous data suggested an association between EBV infection and SLE phenotype presenting with cutaneous and joint involvement, a separate analysis of the relationship between baseline clinical and viral parameters and the achievement of remission and LLDAS in these subgroups of SLE patients was performed [13,14]. The results of univariate and multivariate regression modeling are shown in Table 4. The titer of anti-EA (D) IgM, the presence of anti-EA (D) IgM, and the level of rheumatoid factor were potential, while the titer value of anti-EA (D) IgM was an independent predictor of remission and LLDAS in SLE patients with active mucocutaneous manifestations at enrollment (RR = 2.694, 95% CI RR = 1.04–7.01, *p* = 0.042). Leukopenia and the presence of anti-EA (D) IgM were potential as well as independent predictors of remission and LLDAS in SLE patients with active rash at enrollment (excluding patients with alopecia and mucosal ulcerations only) (RR = 26.313, 95% CI RR = 1.65–973.87, *p* = 0.021, RR = 40.097, 95% CI RR = 1.65–973.87, *p* = 0.023, respectively). Positivity for RF, its concentration, and nonsmoking were potential, while only the positivity of RF was an independent predictor of remission and LLDAS (RR = 12.081, 95% CI RR = 1.48–98.28, *p* = 0.020) in SLE patients with active alopecia. Analysis of subgroups of patients with active musculoskeletal and hematological manifestations, active lupus nephritis, positive anti-dsDNA, anti-Sm, and anti-SSA autoantibodies at enrollment did not reveal any factors associated with achieving remission and LLDAS.

### 2.5. SLE-Related Features as Predictors of Transition from Active to Latent EBV Infection

We investigated whether baseline clinical and immunologic features of SLE patients are associated with a transition from active to latent EBV infection. The presence of lymphopenia, higher ANA titers, and positive anti-dsDNA antibodies was negative, whereas the presence of Hashimoto’s thyroiditis, a higher lymphocyte count, and a higher C3 level were positive predictors of the transition from active to latent EBV infection in a univariate analysis. However, in multivariate regression modeling, lower titers of ANA antibodies and higher C3 levels were independent predictors of a transition to latent EBV infection (RR = 0.996, 95% CI RR = 0.99–0.99, *p* = 0.036 and RR = 26.954, 95% CI RR = 1.45–500.34, *p* = 0.027, respectively) (Table 5).

**Figure 2:**
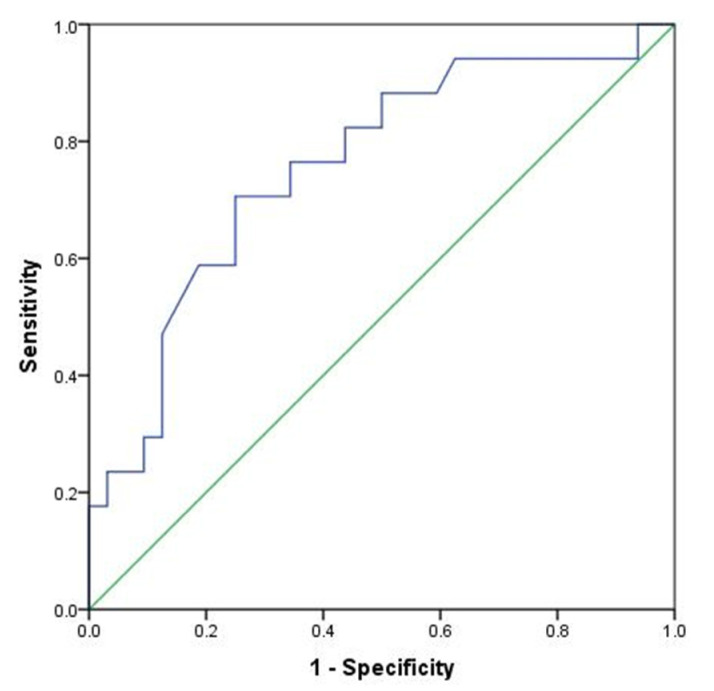
ROC curve analysis of C3 level in differentiating transition from active into latent EBV infection.

We further investigated the possible use of the C3 level as a marker of transition from active to latent EBV infection by performing a receiver operating characteristic (ROC) curve analysis. The area under the ROC curve for the C3 level was 0.756 (*p* = 0.003), suggesting that it could be a useful marker for the identification of patients with active SLE who will transition from active to latent EBV infection. The recommended cut-off value for the C3 level was ≥0.780 g/L (Sn = 70.6% Sp = 75.0%) (Figure 2).

## 3. Discussion

To the best of our knowledge, this is the first study to prospectively investigate the parameters of EBV infection in lupus patients. During the six-month follow-up period, comprehensive serologic and molecular EBV markers were assessed in a cohort of patients with the active form of SLE. The study provided new data regarding the association between EBV reactivation and lupus flares. The high frequency of active EBV infection found at baseline in patients with active lupus declined significantly during follow up, along with a significant decrease in SLE activity. Consistent with the change in EBV infection status, anti-EBNA-1 IgG and anti-VCA IgM titers decreased significantly, whereas anti-EA (D) IgG titers increased during the study. The prevalence of EBV DNA positivity was low at baseline (15.7%) and decreased after follow up, although the difference was not statistically significant. We identified the presence of anti-EA (D) IgM as an independent predictor of remission and LLDAS in SLE patients with a rash. Conversely, a high C3 level was a positive predictor of transition to latent EBV infection in patients with active SLE.

Several previous studies showed an increased prevalence of EBV reactivation compared to controls and an altered humoral response to EBV in lupus patients, but with a wide range of prevalence data described [13,15,16,17,18,19,20]. Similar to our findings, Franca et al. and Han et al. reported a prevalence of active EBV infection of 46.65% (versus 25.92% in patients with rheumatoid arthritis) and 39.66% (versus 10.53% in healthy controls), respectively [14,20]. However, some data suggest a prevalence of up to 78.2% of active EBV infection in lupus patients (versus 62% in controls) [13]. The reasons for these discrepancies are not entirely clear and could be partly explained by the different methods used to determine EBV infection status. Most studies used a limited panel of EBV serological markers, mainly anti-VCA IgM, and/or anti-EA (D) IgG, and/or IgM, and few investigated a comprehensive EBV serological profile in lupus patients [21,22,23]. There is an even greater discrepancy in the data from studies reporting analyses of EBV DNA. For example, a 2019 meta-analysis showed a 55.1% positivity rate for EBV DNA in lupus patients and 20.7% in the control group, but the analysis included highly heterogeneous studies [19]. More recent research from Poland showed that EBV DNA was detectable in peripheral blood monocytes in 74.3% of lupus patients [24]. In contrast, in the study from Turkey, EBV DNA was not found in any sera sample from patients with juvenile SLE [25]. Such inconsistent selection of sample types, methods, and time frames for EBV reactivation testing particularly highlights the problem of the relevance of results interpretation. Considering the aforementioned heterogeneities and limitations, EBV infection status in our study was based on the complete serological profile and/or the presence of EBV DNA, which significantly increased the accuracy of infection status determination.

The triggers and consequences of increased EBV reactivation in lupus patients are not well understood. B-cells abnormalities and intrinsic immune defects associated with SLE result in an impaired immune response to EBV infection allowing repeated reactivations. Several studies have explained the association between EBV reactivation and higher lupus disease activity by linking viral reactivation to increased inflammation and activation of the IFN pathway [15,20,26]. Since most previous studies were cross-sectional or retrospective, the prospective evaluation of EBV markers for a more comprehensive and indepth analysis was obviously lacking. Considering this need, our study included a cohort of patients with active SLE over a six-month period. Most patients with active EBV infection transitioned to latent infection after follow up, as reflected by changes in the serologic profiles and titer levels of the anti-EBV antibodies. However, 22.7% of patients with chronically active EBV infection were also identified. In addition, 11.1% of patients with initially latent infection transitioned to active EBV infection during the follow-up period. Seroprevalence and titers of anti-EA (D) IgM did not change significantly, though we noticed that most of the patients positive after follow up de novo developed these antibodies, indicating a new EBV reactivation. This may suggest that in SLE patients with active disease, frequent EBV reactivations are an important feature of the course of the underlying immune imbalance. Whether these reactivations are influenced by the individual ability to control EBV replication along with the dynamics of lupus activity is unclear. A chain reaction hypothesis has been described, implicating polyclonal B-cell activation during the lupus flare, EBV reactivation, and its further contribution to disease activity [27]. Although in our cohort most patients with active EBV infection transitioned to latent infection, there was no difference in terms of achieving remission at six months. The heterogeneity of the cohort and temporal variations in the flare onset could influence this result. Interestingly, we could not identify any study that prospectively evaluated EBV infection status in lupus patients to compare these results. Further validation of the data in a larger prospective cohort is needed to verify our findings.

The analysis of patients with specific SLE manifestations in this study suggests a possible role of EBV in certain lupus phenotypes. In particular, we report for the first time the possible role of a higher titer of anti-EA (D) IgM as a positive predictor of remission and LLDAS in SLE patients with rash, with an RR = 40.097. To our knowledge, anti-EA (D) IgM antibodies have not been extensively studied in SLE. A 2019 meta-analysis found only three studies examining the seroprevalence of anti-EA IgM antibodies, with an estimated pooled OR of 4.21 compared to controls [19]. EA is a complex of nonstructural proteins encoded by the BMRF-1 gene, early in the lytic cycle. EA (D) acts as a cofactor of viral DNA polymerase, which is required for the initiation of lytic replication. Therefore, anti-EA (D) antibodies are considered a serological indicator of lytic replication [28]. Most literature data focus on molecular mimicry between lupus autoantigens and EBNA1 proteins, though some investigators suspected analogous mimicry with proteins of the EA complex, relying on data from the highlighted anti-EA IgG immune response in SLE [29]. An association between anti-EA (D) IgG positivity and anti-ENA antibodies and Raynaud’s phenomenon, as well as with cutaneous and joint involvement and anti-Ro antibodies, has already been demonstrated [29,30]. A study confirming these assumptions showed that peptides derived from EA and LMP1 increased ANA positivity, anti-SmB, and anti-SmE in mice, whereas EA4 (EA-derived peptide) also increased anti-SmD and anti-Ro antibodies. Moreover, the level of anti-EA4 antibodies correlated with the SLEDAI score, suggesting that they can be used as potential biomarkers of lupus severity [31]. Finally, none of the aforementioned studies examined anti-EA (D) IgM antibodies. Whether anti-EA (D) IgM antibodies mark SLE subsets that are more likely to achieve remission and LLDAS requires further investigation.

We also examined lupus-related predictors of the transition from active to latent EBV infection. For the first time, we demonstrated a higher C3 level as an independent predictor of transition to latent EBV infection. Patients with active SLE and active EBV infection who had higher C3 levels had up to a 26-fold increased chance of transitioning to latent EBV infection. The ROC curve analysis showed that the cut-off value for the C3 level was ≥0.780 g/L with a sensitivity of 70.6% and a specificity of 75% distinguished patients with active SLE who will transition into latent EBV infection. There are several described evidences of association between EBV and the complement system [32,33]. The product of C3 cleavage that occurs upon complement activation is C3d, which binds to the complement receptor CR2 (CD21), also known as the receptor for EBV on B cells. This binding is enabled by significant amino acid sequence homology between the viral glycoprotein gp350 and C3d [34]. EBV and purified gp350 activate the alternative complement pathway [35,36]. Products of C3 and C4 cleavage bind to EBV in an attempt to neutralize viral infectivity and allow binding to CR1 receptors on phagocytic cells. However, purified EBV has been shown to regulate complement activation by acting as a factor I cofactor. Factor I facilitates the cleavage of virus-bound C3b into the inactivated form iC3b as well as the cleavage of C4b into iC4b [36]. This process has been considered another EBV mechanism of immune system evasion. Knowing that SLE patients often have low C3, especially during active disease, this finding could indirectly imply that patients with more active SLE also have greater problems controlling EBV infection. A recent analysis of serologic evidence with a meta-analysis of the association of EBV and Sjögren’s syndrome revealed an inverse association between the titer of anti-EA IgG and C3 and C4 levels [37]. Since anti-EA IgG is considered an indicator of frequent EBV reactivation, the authors suggested that low C3 and C4 levels are the consequence of exhaustion due to EBV-induced activation of the complement system. Given these, and our findings, one could speculate that patients with a higher ability to compensate for increased C3 consumption may be more likely to control EBV infection and transition into latency. If subsequent studies confirm this hypothesis, this could open new therapeutic possibilities for SLE by acting on the complement system.

There are several limitations of the study that need to be acknowledged. Although the number of patients included is relatively small, it should be noted that the cohort consisted only of patients with active SLE. In addition, the six-month period may be too short for some lupus patients to achieve remission, especially in patients with more severe disease. Longer follow up and serial measurements could provide further insight into the delicate relationship between EBV reactivation and lupus activity.

## 4. Materials and Methods

### 4.1. Study Design, Patients, and Samples

A prospective cohort study that included 51 patients with the active form of SLE was conducted at the Clinic of Allergy and Immunology, the University Clinical Center of Serbia, and Institute of Rheumatology in Belgrade, between June 2020 and May 2022. All patients met the internationally accepted classification criteria for SLE, had lupus flare at the time of enrollment, and with SLE Disease Activity Index 2K—SLEDAI-2K ≥ 6 [38,39]. The patients were ≥18 years old and were on a stable prednisone dose (≤20 mg/day) and/or antimalarials for at least 4 weeks and/or immunosuppressants for at least 8 weeks before the first sample collection for virological analysis. Exclusion criteria were a life-threatening form of active SLE, pregnancy, acute infection, and severe hepatic, renal, or cardiopulmonary involvement. A detailed clinical interview and physical examination were performed on all patients, and relevant demographic, clinical, and laboratory data were collected. We used the SLEDAI-2K and physician global assessment (PGA) to assess disease activity [40,41]. Damage accrual was assessed using the SLICC/ACR damage index. Virological tests (anti-EBV antibodies and EBV DNA) were also performed. Six months later, clinical, laboratory and virological reassessment was performed. Since two patients died during follow up, the final analysis included 49 patients. The primary outcome measure was the achievement of SLE remission or lupus low disease activity state (LLDAS) after six months of follow up. Remission was defined as a total SLEDAI-2K score = 0, while LLDAS was defined as a total SLEDAI-2K score ≤ 4, with no activity from major organ systems, and a PGA < 1 [42,43,44]. 

Plasma and serum samples were collected at the time of enrollment (baseline) and 6 months of follow-up and stored at −70 °C until analysis. All samples were collected during active SLE and before increasing corticosteroid and immunosuppression therapy. Written informed consent was obtained from all participants. The Ethics Committee of the Faculty of Medicine, University of Belgrade, approved the study (No. 1550/ IX-14).

### 4.2. Evaluation of Serological Markers of EBV Infection and EBV DNA Detection

The following anti-EBV antibodies were identified and measured in the collected sera using a commercial ELISA kit according to the manufacturer’s instructions (Euroimmun, Lubeck, Germany): anti-VCA IgG and IgM, anti-EA (D) IgG and IgM, and anti-EBNA-1 IgG. For each assay, calibrators were used to calculate the ratio of index values/optical density (OD) to determine the quantitation of IgG antibody levels or semiquantitation of IgM antibody levels. All assays were performed according to predetermined quality control criteria based on positive, negative, and blank controls. IgG antibodies presence was considered positive if values were equal to or greater than 20 relative units (RU/mL). IgM antibodies presence was considered positive if the OD ratio was ≥1.1. Absorbances were recorded on a Multiscan FC microplate reader (Thermo Scientific, Waltham, MA, USA) at a wavelength of 405 nm and background subtraction at 650 nm.

Viral DNA was extracted from 200 μL plasma using a PureLink Genomic DNA Mini Kit (Invitrogen from Thermo Fisher Scientific, Massachusetts, USA) according to the manufacturer’s instructions. One hundred DNA isolates were further used in a nested-PCR procedure to amplify the EBNA1 gene. Amplifications of the C terminus of EBNA1 were performed by nested PCRs as previously described, using primers reported by Lorenzetti et al. [45,46].

Both EBV DNA and anti-EBV antibodies were used to determine the EBV infection status. Patients positive for EBV DNA and/or any anti-EBV IgM antibodies were considered to have active EBV infection.

### 4.3. Autoantibodies and Immunoserological Testing

Serum samples from SLE patients were tested for the presence of the following antibodies: antinuclear (ANA), anti-dsDNA, anti-Sm, anti-SSA, anti-cardiolipin (aCL), and rheumatoid factor (RF). An indirect immunofluorescence (IIF) assay on Hep-2 cells (Aesku Diagnostics, Germany) was used to detect and characterize ANA. ANA titers ≥ 1:80 were considered positive. Antibodies against dsDNA were measured using the Crithidia luciliae IIF assay (Euroimmun, Termophisher, Germany). Anticardiolipin IgG/IgM, anti-SSA, and anti-Sm antibodies were measured by ELISA (Demeditec Diagnostics, Germany). Positive results were considered according to the manufacturer’s instructions. Serum concentrations of C3, C4, IgG, and RF were determined by nephelometric methods (Automatic Biochemistry analyzer Spin 200E-Spinreact) using the manufacturer’s recommended reference values.

### 4.4. Data Analysis

Categorical data were described with absolute and relative numbers. Numerical data were presented as the arithmetic mean with standard deviation or median with IQR/range, depending on the data distribution. Normal distribution was assessed using mathematical (Shapiro–Wilk and Kolmogorov–Smirnov tests, skewness, and kurtosis) and graphical (histogram, boxplot) methods. As this study is part of a larger project, the sample size for the actual objective was not calculated. Instead, the power of the study was estimated using the data obtained for the primary outcome (number of active and latent EBV infections at baseline and after 6 months of follow up). For the two-sided way of conclusion within the McNemar test for the dependent sample, an α-value of 0.05, a total sample size of 51, a calculated odds ratio of 0.18, and a proportion of discordant pairs of 0.4, the estimated power of the study was 91.17%. McNemar’s test was applied to compare dichotomous data in the dependent sample. Wilcoxon’s signed rank test was used to assess differences in numerical data without normal distribution at baseline and after 6 months of follow up. To evaluate predictors of remission in SLE patients at 6 months of follow-up, first univariate, then multivariate logistic regression analysis was performed adjusting for age, sex, and lupus therapy, reporting the risk ratio (RR), 95% confidence interval of the risk ratio (95% CI RR), and *p*-value. The receiver-operating characteristic curve (ROC) was used to determine the cut-off value of C3 that can discriminate patients transitioning from active to past EBV infection at 6 months.

## 5. Conclusions

In conclusion, this is the first study to prospectively investigate the dynamics of serological and molecular markers of EBV infection in SLE patients. The results showed frequent EBV reactivation in patients with active SLE, but most of them transitioned to latency after 6 months. Transition to latent EBV infection was significantly more likely in patients with higher C3 levels. We also provide the first evidence for the potential use of anti-EA (D) IgM antibodies in stratifying lupus patients and predicting the outcome in patients with cutaneous involvement. Further data demonstrating a specific link between EBV and SLE may provide deeper insight into new targeted lupus therapies.

## Figures and Tables

**Table 1:** Characteristics of the study population during the follow up.

Characteristic	Baseline	Follow-Up	*p* Value
Clinical manifestations			
Rash, n (%)	29 (56.9)	15 (30.6)	0.001
Arthritis, n (%)	34 (66.7)	13 (27.1)	<0.001
No. of tender and swollen joints, med (IQR)	4 (0–6)	0 (0–1)	<0.001
Mucosal ulcerations, n (%)	11 (21.6)	2 (4.2)	0.021
Alopecia, n (%)	29 (56.9)	16 (32.7)	<0.001
Serositis, n (%)	11 (21.6)	1 (16.7)	1.000
Active lupus nephritis °, n (%)	15 (29.4)	6 (12.5)	0.004
Active NPSLE, n (%)	3 (5.9)	0 (0.0)	1.000
Leucopenia, n (%)	11 (21.6)	5 (10.2)	0.180
Neutropenia, n (%)	9 (18.8)	6 (12.5)	0.727
Lymphopenia, n (%)	33 (67.3)	27 (56.3)	0.180
Thrombocytopenia, n (%)	8 (15.7)	4 (8.2)	0.125
Immunological parameters			
ANA titer, med (min-max)	640 (80–640)	640 (80–640)	0.185
anti-ds DNA positivity, n (%)	19 (37.3)	17 (35.4)	1.000
anti-SSA positivity •, n (%)	27 (54.0)	NA	
anti-Sm positivity •, n (%)	19 (37.3)	NA	
aCL IgM positivity •, n (%)	7 (14.0)	NA	
aCL IgG positivity •, (%)	8 (16.0)	NA	
low C3 and/or C4, n (%)	41 (80.4)	21 (42.9)	<0.001
C3 (g/L), med (min-max)	0.71 (0.10–1.4)	0.90 (0.30–1.43)	0.002
C4 (g/L), med (min-max)	0.08 (0.02–0.87)	0.15 (0.02–2.40)	<0.001
RF positivity, n (%)	14 (28.6)	9 (18.4)	0.227
IgG (g/L), med (min-max)	13.50 (4.4–31.6)	12.80 (4.50–22.30)	0.054
Inflammatory markers			
ESR (mm/h), med (min-max)	30.00 (4.00–125.00)	20.00 (2.00–90.00)	0.01
CRP (mg/L), med (min-max)	4.04 (0.20–87.5)	2.20 (0.01–41.60)	0.039
Disease activity			
SLEDAI 2K, med (IQR)	10.00 (6–29)	4.00 (0–16)	<0.001
clinical SLEDAI 2K, med (IQR)	8.00 (6–10)	3.00 (0–5)	<0.001
PGA, med (IQR)	1.38 (0.75–2.34)	0.66 (0–1.86)	<0.001
SLE therapy			
Prednisone (daily dose mg), med (min-max)	20.00 (5–80)	10.00 (2–30)	0.001
Immunosuppressive therapy (AZA, MTX, MMF, CYC) *, n (%)	16 (31.7)	30 (63.8)	<0.001
SLICC/ACR Damage Index, med (min-max)	0.00 (0–6)	1.00 (0–4)	0.003

* AZA—azathioprine; MTX—methotrexate; MMF—mycophenolate mofetil; CYC—cyclophosphamide. °—defined as 24 h-proteinuria > 0.5 g and/or active urine sediment. •—measured only on initial evaluation.

**Table 2:** EBV infection status during the follow up.

EBV Infection Status, *n* (%)	Active at Baseline (*n* = 22)	Latent as Baseline (n = 27)
Active at follow up (*n* = 8)	5 (22.7)	3 (11.1)
Latent at follow up (*n* = 41)	17 (77.3)	24 (88.9)

**Table 3:** Seroprevalence of anti-EBV antibodies during the follow-up.

anti-EBV Abs, *n* (%)	Baseline, *n* (%)	Follow-Up, *n* (%)	*p* Value
anti-EBNA-1 IgG	50 (98)	42 (85.7)	*p* = 0.031
anti-VCA IgG	51 (100)	49 (100)	NA
anti-VCA IgM	16 (31.4)	2 (4.1)	*p* < 0.001
anti-EA(D) IgG	21 (41.2)	24 (49.0)	*p* = 0.227
anti-EA(D) IgM	10 (19.6)	6 (12.2)	*p* = 0.388

**Table 4:** Factors associated with remission and lupus low disease activity state (LLDAS) in SLE patients with active mucocutaneous manifestations at enrollment.

Factors Associated with Remission/LLDAS in:	Univariate Logistic Regression			Multivariate Logistic Regression		
	RR	95%CI RR	*p*	RR	95%CI RR	*p*
Mucocutaneous manifestations (*n* = 40)						
Titer level of Anti-EA(D) IgM ^b^	2.875	1.12–7.38	0.028	2.694	1.04–7.01	0.042
Presence of anti-EA(D) IgM ^b^	6.650	1.16–38.19	0.034			
RF ^b^	1.026	0.99–1.05	0.049	1.027	0.99–1.06	0.110
Rash ^b^ (*n* = 29)						
Leucopenia ^b^	10.714	1.05–109.78	0.046	26.313	1.65–419.27	0.021
Presence of anti-EA(D) IgM ^b^	10.714	1.05–109.78	0.046	40.097	1.65–973.87	0.023
Alopecia ^b^ (n = 29)						
Smoking status ^b^	0.143	0.02–0.83	0.031	0.242	0.03–1.96	0.184
Positive RF ^b^	11.375	1.65–78.38	0.014	12.081	1.48–98.28	0.020
RF concentration ^b^	1.057	1.01–1.11	0.019			

^b^—at baseline.

**Table 5:** Predictors of transition from active to latent EBV infection in 24 SLE patients with active EBV infection at enrollment.

Variable	Univariate Logistic Regression			Multivariate Logistic Regression Backward Wald Method 4th Step		
	RR	95% CI RR	*p*	RR	95% CI RR	*p*
Lymphopenia ^b^	0.227	0.06–0.83	0.025			
Hashimoto thyroiditis ^b^	6.042	1.03–35.55	0.047			
Number of lymphocytes ^b^	2.315	1.12–4.80	0.024			
ANA titer ^b^	0.996	0.99–0.99	0.019	0.996	0.99–0.99	0.036
Anti-dsDNA Abs presence ^b^	0.214	0.05–0.89	0.034			
C3 level ^b^	36.441	2.48–536.32	0.009	26.954	1.45–500.34	0.027

^b^—at baseline.

## Data Availability

Data are available upon reasonable request. All data relevant to the study are available on reasonable and justified request. Please contact the corresponding author. They are not publicly available to ensure the strict confidentiality of the patient’s data.
